# Keeping Up With School During Hospitalization for Children With Chronic Illnesses, Siblings and Parents

**DOI:** 10.1111/cch.70161

**Published:** 2025-10-06

**Authors:** Margaret Wazevich, Riley Justice, Nneka Bonner, Josheph Lampton, Laura Nabors

**Affiliations:** ^1^ School of Human Services, College of Education, Criminal Justice, Human Services, and Information Technology University of Cincinnati Ohio Cincinnati USA

**Keywords:** children with chronic illness, hospitalization, parents, school, siblings

## Abstract

**Background:**

Children who have chronic illnesses (CIs) and their siblings often miss school when the child with a CI is hospitalized. Understanding the perspectives of the child with an illness, siblings and parents will provide information about how they keep up with schoolwork, their perceptions of not being in school, or of homeschooling that will inform educators, families and medical professionals.

**Methods:**

English‐speaking children diagnosed with CI, siblings and parents participated. They were residing at a Ronald McDonald House. A qualitative study using interviews with children with CIs, siblings and parents was completed.

**Results:**

All three informant groups reported positive experiences with completing schoolwork, and teacher flexibility and availability were key to child success. Ensuring that schoolwork covered the basic academic subjects and materials was important, as children were tired and could lack time to complete work. Missing friends and school, and after‐school activities were negative effects of missing school. Several parents homeschooled their children and felt positively about this experience, as they were then able to care for their child's medical needs, match educational lessons to their child's abilities and ensure they had time to make the child's medical appointments.

**Conclusions:**

Children with illnesses, siblings and parents reported that their school experiences during the hospitalization of the child with an illness were usually positive. Importantly, children were able to keep up with their work. Children did miss interacting with teachers, peers and engaging in school and after‐school activities. In the future, more information about the quality and impact of homeschooling will provide information to advance knowledge on ways to support the academic needs of children who are educated at home.

## Summary

- Children with CIs completed their schoolwork during hospitalization, which promoted a sense of normalcy.
- Teacher flexibility and availability assisted parents, who were typically serving as tutors for their children with CIs and siblings.
- Parents who homeschool their children may do so because they feel they are better equipped to care for their child's medical needs than the professionals in schools.
- Children with CIs and siblings missed social interactions with their friends and participation in school activities during hospitalization.

## 1 Introduction

Chronic illnesses (CIs) involve physical, behavioural, developmental or mental health conditions lasting more than 3 months and typically require hospitalization (Baker and Claridge 2022; Mokkink et al. 2008). Although rates vary, about 10%–20% of children have CIs (Baker and Claridge 2022; Kohl and Barnett 2020). Children with CIs often have difficulty attending school regularly, in part due to hospitalizations as well as medical appointments (Barnett et al. 2020; Hu et al. 2022; Wikel and Markelz 2023). Hospitalizations disrupt both academic progress as well as the child's sense of normalcy with the flow of everyday life and social connections with peers (Hu et al. 2022). If children miss school and fall behind academically due to their medical conditions, it can negatively affect their emotional well‐being (Bravo et al. 2020). Ciucci et al. (2024) reported that children who continued their schooling and completed academic work during hospitalization showed improved mood and experienced lower levels of pain, which can be important to recovery. Continuing schooling while in the hospital can promote normalcy for children, as they are able to continue a critical daily routine (Dinç et al. 2023).

Parents value educational support while their child is in the hospital (Delloso et al. 2021). Some children have access to schooling available from the hospital, which could be in the form of going to hospital‐school classrooms or having access to teachers who work at the hospital and can tutor children (Barnett et al. 2020; Caggiano et al. 2021; Dinç et al. 2023). However, many parents and children may not have access to this type of support, and not all parents wish to access these services. Therefore, many children rely on their parents for academic support, which can be an added stressor for parents (Delloso et al. 2021). In turn, parents may value and rely on support from teachers from their child's school while their child is in the hospital (Baker and Claridge 2022). Less is known about facilitators and barriers for this informal educational support during hospital stays and how parent–teacher and teacher–child connection is working to support academic success. Additionally, during hospital stays, children are separated from their peers and the social and psychological support of teachers (especially when there is not a hospital school), which may be a source of stress for them (Caggiano et al. 2021). Parent and child perspectives on teacher behaviours that support progress will also provide guidance for working with children and parents during a very vulnerable period in the illness course.

There is a body of research on school reintegration programs after hospitalization, and this research shows how important connecting with the medical team and developing individualized programming to support school success can be to support the child (Carlton et al. 2021; Leite et al. 2023). These programs have been successful in helping children navigate the transition to school and in identifying specialized programming to help them once they return to school (Canter and Roberts 2012). However, some parents may not prefer school reintegration or having their children attend the school in their community. Some may have concerns that the school cannot care for their child's complex medical needs (Morales Ruíz et al. 2025; Uhm and Choi 2020). Some children with complex needs could be reticent to return to school—either fearing their medical needs could not be met, feeling that their complex care and condition would not appear ‘normal,’ or they would draw too much attention in a regular school setting (Uhm and Choi 2020). During hospitalization, through care at home, and interactions with the medical team, parents become experts on their child's medical treatment (Baker and Claridge 2022). They may feel uncomfortable coordinating the care of their child and coordinating the sharing of information and may prefer to care for and provide schooling to their child with an illness, and his or her siblings, at home (Miller et al. 2009). Information is needed about children's and parent perceptions of homeschooling when there is a child with a CI in the family.

Over 250,000 siblings are living with a brother or sister with a CI (Mooney‐Doyle et al. 2022). Siblings often spend time with the child while he or she is hospitalized, and with their parents being away more often, they too can experience disruption in their normal routines. Siblings need support to cope with their brother or sister's illness and the family stress (Deavin et al. 2018). When children with CIs are hospitalized, it impacts the functioning of siblings, and although they often cope quite well and do well in school, they may experience significant stress (Keller et al. 2023). Gan et al. (2017) reported that siblings are at risk for poor school attendance and difficulties with completing work and academic achievement. They also may feel differences in or a lessening of support from teachers and peers. They called for future research to understand siblings' school experiences. Interactions with peers may be especially supportive for siblings, to help them take a break from stress and worry over their sibling's CI (Gan et al. 2017). The current study examined siblings' perceptions of their schooling while their brother or sister was hospitalized or recovering from hospitalization.

Rolland (1987) proposed that CI are interconnected with the systems in which an individual is situated including the parent–child unit, child–sibling unit, family, hospital, and school. Similarly, Walsh (2003) stated that a CI touches all family members, and thus it is important to examine multiple perspectives when designing interventions (Huth et al. 2023; Walsh 2003). As such, it is critical to understand parents' and children's perspectives of how the family cope with school during events, like hospitalization, that disrupt their typical school experience. Gaining an understanding of parent beliefs about their children attending school in the community also facilitates knowledge for integration of children with illnesses in traditional school settings. Understanding family views, strengths, and stressors can improve education in the hospital and beyond, which is essential to child development, as school experiences play a role in shaping child social and academic development (Law et al. 2013; Patterson 2002; Roeser et al. 2000). The purpose of the current study was to understand perspectives of academic needs and missing school reported by children with CIs, their siblings and parents when the child was hospitalized. Understanding the needs and coping of the family while the child is hospitalized can inform plans for academic continuity, which is critical to academic success and school reentry (Thompson et al. 2015; Wilkie 2012). The current study involved children with a variety of CI, and this noncategorical approach provides a broad view of how children with CIs handle academic progress during hospitalization (Stein and Jessop 1982). Including siblings was important, as they miss school as well, when they accompany the family. Interviews were used to assess their perceptions of the difficulties with completing schoolwork, supports for completing schoolwork, connection with their teachers while hospitalized and their views of barriers and facilitators related to missing school while in the hospital. Learning about family experiences and perspectives can help provide insight on how to best support students with CIs and their siblings with academic and social needs related to being absent from school due to hospitalization of a child with CIs in the family.

## 2 Methods

### 2.1 Study Design and Participants

This qualitative study used two convenience samples of parents and children. There were two phases to the study: data collection (Phase 1) and a member check (Phase 2). A university‐based institutional review board approved this study.

#### 2.1.1 Phase 1: Interviews

In Phase 1, children with CIs, their siblings and parents completed interviews. They were residing at a RMH while the child with an illness was recovering from hospitalization or undergoing hospital procedures, and they were English speakers. Participants were 14 children with CIs, 10 siblings and 31 parents (25 mothers and 6 fathers).

#### 2.1.2 Phase 2: Member Check

A different group of five children with CIs, three siblings and eight parents (seven mothers and one father) who spoke English participated. Their role was to verify the themes presented in Phase 1. They completed two tasks. First, parents, children with CIs and siblings learned about the interviews and questions used in Phase 1. Then, they reviewed themes for interview questions to determine if themes represented their experiences. These participants were a different group, albeit in the same population as those participants from Phase 1.

### 2.2 Data Collection

#### 2.2.1 Phase 1: Interviews

Parents provided consent and children provided assent. M. W., J. L., N. B. and L. N. conducted interviews and transcribed information from their interviews. Data were collected over 12 scheduled dates.

Parents provided demographic information about their child, and they completed interviews. Interview questions for parents were: (1) What's ‘out of school’ like for your child when they are in the hospital? (2) What does your child need to help them catch up on school assignments? (3) What motivates your child? (4) What, if any, factors hinder or make it harder to catch up on work? (5) What do you think is the hardest part about your child not being in a traditional school setting? (6) What are your child's academic needs? Probing question: How are they addressed? (7) If your child does not attend a traditional school, what type of school setting do they receive their educational needs (home school, alternative school)? Probing questions: (a) If your child is homeschooled, do you provide the lesson planning, or do they participate in online lessons? (b) If your child is homeschooled, how does being ‘out of school’ affect their learning outcomes (such as catching up on online work, attendance, affect lessons)?

**FIGURE 1:**
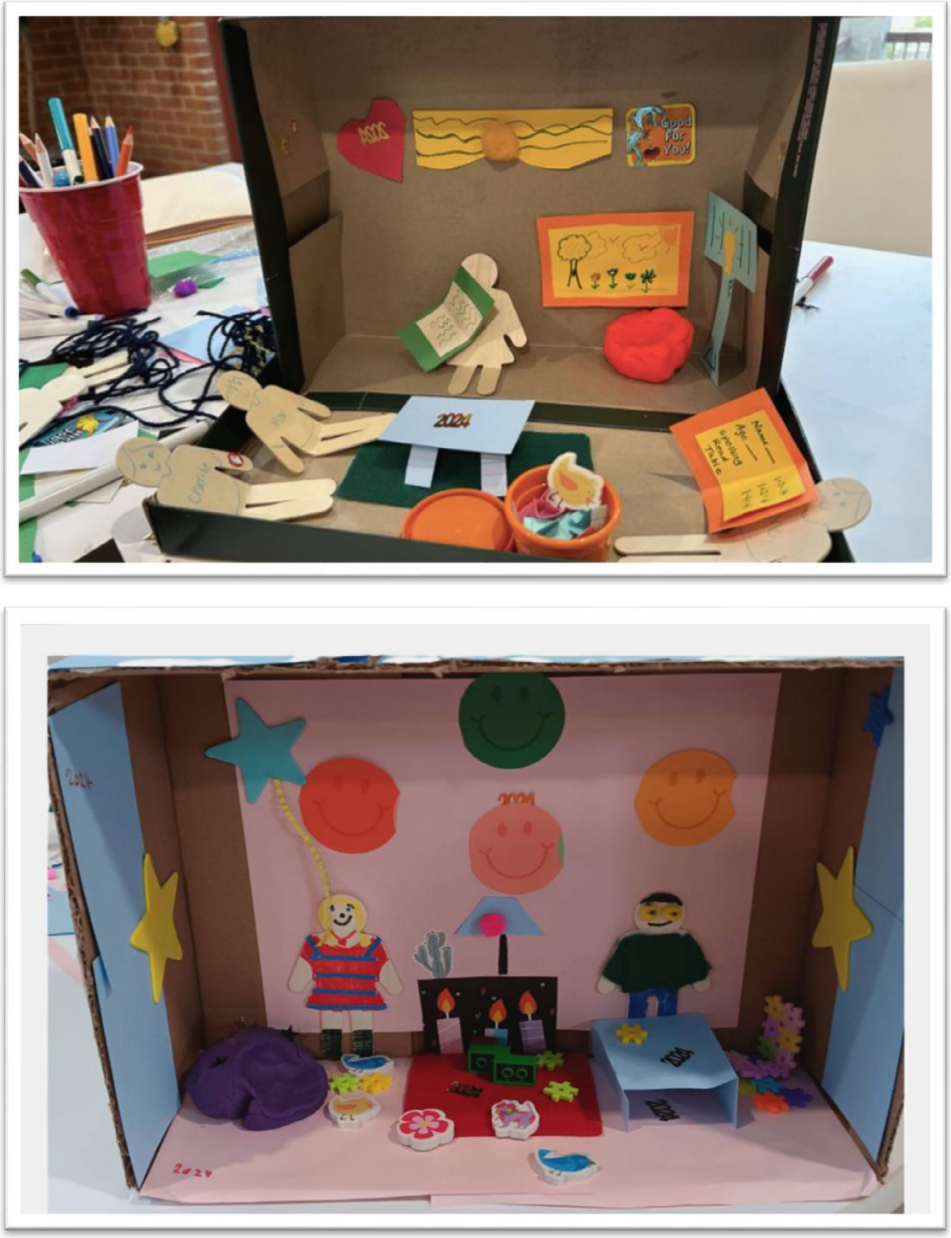
Dioramas depicting ‘school outside of school’ while the child was in the hospital.

Children with CIs and siblings developed a diorama, using a shoebox and art materials to represent their perception of school while in the hospital (see Figure 1).

The prompt for the art activity was to make a room that shows your school outside of school while you are at the hospital. Nabors and Liddle (2017) previously used a diorama method with children to discuss children's perceptions of being in the hospital. For the current study, the diorama was an artefact to refer to (a cue to discuss schooling while the child with a CI was at the hospital), when discussing the interview questions. While completing their dioramas and/or right after finishing them, children with CIs and siblings completed interviews. Interviewers helped the child with their artwork as needed, and they used the diorama as a prompt to think about schooling at the hospital, as children completed the interview questions (Nabors and Liddle 2017).

The questions were the same for children with CIs and siblings: (1) What is your school like when you are at Ronald McDonald House? Probing questions: Who helps you? What do you get help with? Does the teacher visit to help online? (2) How is your schoolwork at Ronald McDonald House different from schoolwork in the classroom? Probing questions: What is good about it? What is not good about it? What would make it better or easier to do your schoolwork? (3) What is it like to do schoolwork outside of the classroom without a teacher? Probing questions: Is it harder? Easier? What is it like to work on your own and not with your class? How do you feel about being outside of school or not in school? (4) What about school do you miss when you are not there? Probing questions: Who do you miss? Why? How do you keep up with your friends from school when you are not there? (5) Do you miss specials? Probing questions: Which one? Why? (6) Do you miss your teacher(s)? Probing question: Tell me more about this. (7) Do you miss after school activities? Probing questions: Which one(s)? How do you feel about this? The children's responses to interview questions provided the qualitative data that was analyzed to understand their perspectives.

#### 2.2.2 Phase 2: Member Check

A different group of parents and children, with CIs and siblings, who were at the same Ronald McDonald House, learned about the interview questions and then reviewed themes for answers provided by children with CIs, siblings and parents. Thus, after learning about the interview questions and then the responses provided by the first group of children with CIs, the children with CIs in the member check verified themes (reported as to whether the themes reflected their experiences and were accurate) and were offered a chance to add new information to answer the questions. Siblings completed a similar procedure—reviewing the interview process and answers provided by siblings who participated in Phase 1. They were asked if the themes represented their experience and were invited to present new information if they wished to do so. The same type of process was followed for parents in the member check group.

L. N., M. W., N. B. and R. J. presented the information to parents and children and recorded their responses. The interview questions were presented with a list of themes determined from the analyses for Phase 1. Parents and children could agree or disagree with the themes or add new information to answer the questions. Data were collected over two meetings. This method has been used in a previous study to audit and verify themes about hospital experiences for parents and children (Nabors and Liddle 2017).

### 2.3 Analysis

#### 2.3.1 Phase 1: Interviews

R. J., L. N. and H. F. independently reviewed the interview transcripts using an open coding process with memos to determine common codes and themes in the data (Braun and Clarke 2006; Creswell and Poth 2023; Miles and Huberman 1994). R. J., a graduate student, and L. N., a psychologist, had experience working in schools with young children who have health conditions, and H. F. had experience working with children. H. F. was an undergraduate health sciences major attending a different university who assisted with this project for internship credit. H. F. served as the verification coder, and she reviewed the transcripts and audited the coding (Creswell 1998; Meyrick 2006). After developing a list of common themes (i.e., determining their list of codes) in the data, coders found representative quotes for themes. Coders verified the themes and the presentation of the results over three 60‐min meetings. During these meetings, the coders also determined data saturation. Disagreements were resolved by consensus.

#### 2.3.2 Phase 2: Member Checks

L. N., R. J. and H. F. reviewed data to determine which themes were endorsed by parents and children and to determine if new information was added to answer the interview questions.

## 3 Results

### 3.1 Phase 1: Interviews

Demographic data for Phase 1 is presented in Table 1. The demographic homogeneity of the sample is evident in Table 1 and influences the generalizability of findings.

**TABLE 1:** Demographic information for parents, children with CIs and siblings.

Participant group	Variable: categories	*n*	*M* (range)
Parents	Marital status:		
	Married	19	
	Divorced	2	
	Unknown	10	
	Sex:		
	Male	6	
	Female	25	
	Ethnic group:		
	White	29	
	Biracial	2	
	Age:		38 years, 5 months (30–63)
Children with CIs	Sex:		
	Boys	4	
	Girls	10	
	Ethnic group:		
	White	10	
	Biracial	4	
	Age:		7 years, 9 months (6–13)
	Primary diagnoses:		
	Autism	1	
	Eosinophilic esophagitis	1	
	Rett syndrome	1	
	Foot deformity	1	
	Chronic pancreatitis	1	
	Tethered spinal cord	1	
	Inflammatory disease	1	
	22Q	1	
	Chiari malformation	1	
	Laryngomalacia	1	
	Burkitts lymphoma	1	
	Gomez–Lopez–Hernandez syndrome	1	
	Brain tumour	1	
	Idiopathic scoliosis	1	
	Myofibrillar myopathy	1	
	Metastatic neuroblastoma PCOS	1	
	Genetic mutation	1	
Siblings	Sex:		
	Boys	3	
	Girls	7	
	Ethnic group:		
	White	10	
	Biracial	0	
	Age:		9 years, 8 months(8–14)

*Note:* The demographic skew of the data limits transferability, especially in marginalized populations.

Coders found the following three main areas for themes: (1) positive experiences with completing schoolwork while hospitalized, (2) feelings of loss related to hospitalization and (3) perceptions of homeschooling. These themes were endorsed by parents, children with CIs and siblings.

Table 2 presents themes and subthemes, with representative quotes, for the first area, positive experiences with schoolwork.

**TABLE 2:** Positive experiences with school work during hospitalization for parents, children with CIs and sibling informants.

Themes	Explanation of theme	Representative quotes from parents, children with CIs and siblings
Benefits to being out of school to do academic work	Working independently at my own pace	P: ‘My son catches up quick’ CI: ‘I can catch up quick and focus better on my own’ S: ‘I like doing homework at the Ronald McDonald House without a teacher. It goes quicker.’
Teachers are flexible	Can reach them easily with questions	P: ‘Homeschool provides flexibility to work around medical appointments or therapy.’ CI: ‘Teachers and school staff communicate effectively when away’ S: ‘My teachers are great’
Supportive environment (school was supportive of child and his or her needs)	The school is willing to meet the child's needs related to his or her chronic illness	P: ‘Teachers and school staff communicate effectively when son is away’ CI: ‘My teachers are supportive’ S: ‘Do you [sibling] ever reach the teacher? … I email her’

The themes included, (1) benefits to being out of school to do academic work, (2) teacher flexibility to do the work and (3) perceptions of the school staff being supportive of the needs of the child. For Theme 1, children with CIs and their siblings benefited from being able to work at their own pace, so that they could keep up with their schoolwork. Theme 2 revolved around teacher flexibility, which was high, as participants stated teachers were easy to reach, typically via email. Most participants reported that school staff worked hard to meet the needs of children with CIs and siblings when they had to miss school (see Table 2). Absences from the classroom, especially extended ones, remained problematic, with children feeling distress related to not being in the classroom, missing friends and missing instruction about homework from their teachers. Although parents were great tutors, a few children really missed learning from their teachers in their classrooms, and these ideas are captured in Table 3, which discusses feelings of loss related to a hospital stay.

**TABLE 3:** Feelings of loss due to hospitalization for parents, children with CIs and siblings.

Themes	Explanation of theme	Representative quotes from parents, children with CIs and siblings
Missing activities during the school day	Missing participation in specials and interactions with their friends during the school day	P: ‘Missing his friends… missing extracurriculars’ CI: ‘Miss friends and being physically active with peers’ S: ‘Miss seeing my friends when at RMH’
Negative consequences of absences	Being absent is stressful (i.e., lack of explanation of assignments, loss of instruction time)	P: ‘No accommodations for missing school due to appointments’ CI: ‘Coming back after being away [due to medical appointments/therapy] is stressful.’ S: ‘Attendance suffers; stressful’
Loss of support from teachers and other service providers	Loss of contact with teachers and providers of support services (reading teacher, speech and language, OT/PT)	P: ‘Lack of support when out of school’ CI: ‘My teachers support me, but there's a lack of support when out of school’ S: ‘I do not have a teacher at Ronald McDonald’

The second area for themes was feelings of loss related to hospitalization. Table 3 presents the following three main themes in this area: (1) missed activities during the school day, (2) negative consequences of absences and (3) loss of support from teachers and other service providers. All three informant groups reported that children were missing their typical school and after‐school activities and missing interactions with friends that are a part of these experiences. Parents reported that their children with CIs could experience a sense of loss due to frequent hospitalization, which could lead to feelings of loneliness and anxiety about missing school‐related activities. A few of the parents and children with CIs shared frustration about their school experiences, related to a lack of or poor communication with teachers and school staff not ‘understanding’ how the child's illness influenced academic performance, and teachers not knowing how to manage the child's medical condition in a classroom setting. Missing school activities, loss of support from teachers and missing social interactions with peers from school were stressors for children and the family for educators to consider during the hospitalization of the child with CIs.

Children missed socializing with their friends during the school day as well as during school‐ and community‐based extracurricular activities (see Table 3). As mentioned, although children felt support in catching up on work, they missed explanations of work from the teachers. Children who were educated in school wanted to be in school, learning alongside their peers. Several parents noted that attendance suffers in schools where attendance is heavily emphasized. Children also missed their teachers and ‘special services,’ which included a broad range of services provided by many different types of specialists (e.g., reading teachers, physical therapists). Children discussed having a special relationship with teachers and missing their support. Although communication from the teachers and others at school was usually acceptable, when this was not working, parents and children could feel stress. It could limit opportunities to learn, and for those rare times when communication was poor, parents and children were exasperated and parents could become disenchanted with formal education, believing it did not meet their child's needs.

Area three focuses on homeschooling experiences (see Table 4). The number of parents homeschooling (71%, *n* = 22) may be unique to our sample (parents able to enrol and stay at a local RMH).

**TABLE 4:** Homeschooling experiences for parents, children with CIs and siblings.

Themes	Explanation of theme	Representative quotes from parents, children with CIs and siblings
Positive factors	Parents can choose what to teach and it allows for tailoring of lessons to child needs and abilities	P: Parent reports, ‘… being out of school enhances their [students] learning, having the 1:1 with her is helpful, along with taking multiple breaks.’ CI: ‘[My] parents can help more when I am in the hospital than when I am in a traditional school setting.’ S: ‘[Parent] can bring work materials for me and my sister to keep us up with classwork.’
	Flexibility around the medical appointments and symptoms	P: ‘During the child's stay [at RMH], it was flexible and fine with him being pulled for different appointments to help with his illness.’ CI: ‘My parents are helpful when catching me up with work.’ S: ‘Most of the work she [sibling with CIs] needs is online so she can catch up’
	I do not have to worry that my child's illness needs will not be met if I am the teacher	P: ‘Child would require too many medical needs at school; Medical needs hinder education’ CI: ‘Homeschooling is most supportive for my needs’ S: ‘[Parent] can bring work materials for me and my sister to keep us up with classwork.’
Negative factors	Lack of services (difficult to get special services)	P: ‘Lack of supportive services; Lack of accommodations’ CI: ‘Homeschooling has a lack of services’ S: ‘Difficulties getting extra help [Literacy support]’
	Minimal opportunities for peer interaction	P: ‘Not being around his friends and not being around other kids in general.’ CI: ‘The child reports missing her friends and hanging out with them.’ S: ‘Sometimes miss hanging out with my friends on the playground if we are at the hospital.’

Parents typically thought home schooling was positive for their children (see Table 4). They did not report difficulties with fulfilling their role as teachers. Parents reported that they could teach to meet the medical needs of their child with CIs if they are homeschooled. For instance, if their child needed frequent rest periods due to his or her CI, parents felt that they could provide ‘rest breaks’ on days when their child was tired or in pain. Conversely, they could teach more material on days when their child was not fatigued or in pain. If a child faced cognitive issues, such as a limited attention span, parents also felt that they could adjust lessons so that their child had frequent breaks. One parent stated, ‘being out of school enhances their [students] learning, having the 1:1 with her is helpful, along with taking multiple breaks.’ Several of the parents mentioned they thought homeschooling provided a better educational experience for their child because, as teachers, they could find lessons that ‘fit’ with their child's interests so that he or she would engage with the material.

Parents reported a few negative factors related to homeschooling (see Table 4). Some of the parents mentioned that they were homeschooling because they felt that the traditional school setting could not accommodate the needs for special medical care related to their child's illness (e.g., following medication regimens). A significant downside noted by parents is the loss of access to specialized support services typically available in traditional school settings, such as literacy interventions, speech and language therapy and physical and occupational therapy. For instance, some stated that a lack of support services was related to their decision to homeschool their children. A few parents shared that high levels of absenteeism could be a source of stress, as children miss critical instruction and often face difficulties in catching up on assignments, but most parents stated their children could keep up with assignments. Parents noted that homeschooling can limit opportunities for peer interaction and participation in extracurricular activities, such as sports and special classes.

Most of the children with CIs and siblings who were homeschooled were positive about this experience, reporting that they really enjoyed working on things they liked to learn about with their parents (see Table 4). A few children mentioned that it could be difficult to have a parent as a teacher, but these children said their parents learned to be good teachers. Children with CIs and siblings felt like that they could learn things that were of interest to them—learn their favourite subjects in creative ways. Children with CIs reported that when they were homeschooled, they did not have to worry about becoming tired and not completing work, because they could take breaks and finish work when they had the energy to do so. Being able to do work when one has energy to complete assignments was synonymous with perceptions that homeschooling was an easier way to attend school for children with CIs. Siblings did not report issues with energy levels.

In terms of negative outcomes, children with illnesses and siblings could ‘long for’ social experiences with peers and friends. The lack of these opportunities may hinder a child's ability to form meaningful social connections and engage in the broader school community. Exploring needs in homeschooling situations will allow educators and healthcare professionals to learn of areas for providing resources. Children with CIs noted difficulties in getting extra services (like occupational therapy) they needed when they were homeschooled. Some siblings discussed needing extra help on some assignments that they might get from teachers or other aides in the school setting and mentioned that not getting this support was a downside to homeschooling.

### 3.2 Phase 2: Member Check

Eight parents (7 mothers and 1 father; 7 were White and 1 was African American, age range 30s to early 50s), five children with CIs (4 females and 1 male; all were White, and their ages ranged from 5 to 12 years), and three siblings (all were females; 2 were White and 1 was African American, ages ranged from 6 to 14 years) participated. Medical conditions for children with CIs included: liver transplant recipient, asthma, eosinophilic esophagitis, diaphragmatic hernia, tracheal obstruction, lung disease, aortic stenosis, cancer, and intellectual delay. Children with CIs had more than one diagnosis. Four of the parents indicated they were homeschooling their children, and the other four parents had enrolled their children in school.

Parents endorsed the themes from the interviews—checking all the themes on the checklist provided to them, and they provided additional interview information as they were completing the checklists. Specifically, all parents were positive about their children's progress on homework while at the Ronald McDonald House. Parents of children in community schools confirmed that teachers were accessible via texting, email or online meetings on Zoom/Microsoft Teams. Teachers' flexibility in reducing the length of assignments (to the basics, like important reading and math assignments) and in responding to questions helped their children complete work and remain positive about doing their schoolwork. Parents (*n* = 4) who were homeschooling were positive about this method, and they confirmed that homeschooling occurred because they could schedule learning around their child's medical appointments, and they also felt that they could care for their child's chronic condition better than school staff (i.e., school might not understand how to care for the child's illness). Parents who were homeschooling also confirmed that homeschooling allowed them to tailor the lessons to their child's interests. Parents reported that their children (both those with CIs and siblings) were missing friends at school or in their neighbourhoods, were missing their involvement in extracurricular activities, and could experience loneliness when their child with a CI was hospitalized.

Children with CIs and siblings endorsed all the themes on their checklists. For instance, they confirmed that they were ‘keeping up with schoolwork’ while at the RMH, and their teachers were accessible to help and provide information, and parents were helpers, ensuring that they understood their work. They were satisfied with support from teachers in terms of responding to questions and were relying on their parents as substitute teachers or tutors. They reported that they were missing friends (from school and their neighbourhoods) and were missing their involvement in their usual activities (e.g., sports and other extracurricular activities). Several mentioned that they could keep up on their own with minimal help from parents and teachers. One child mentioned that school reintegration could be difficult after extended absences. Hence, the member check confirmed themes provided by children with CIs, siblings and parents in Phase 1, providing verification of the findings for the primary phase of our study (Creswell 1998; Creswell and Poth 2023).

## 4 Discussion

Results of the study were positive, indicating that children and parents felt teachers from their community schools were supporting children with CIs and siblings while the child with an illness was hospitalized. Children were feeling a loss of their normal routines, especially if they were participating in community‐based schools, and they were missing their friends and teachers. This experience is reflected in other literature (Baskaran et al. 2022; Byhoff et al. 2022; Miao et al. 2024; Spencer et al. 2023). A subset of parents homeschooled their children, typically so they could care for the child's medical needs or deliver the medical regimen. This reason was consistent with other studies where parents felt responsible for the medical regimen (Baker and Claridge 2022; Nygård and Clancy 2018). Parents who homeschooled their child said an advantage was that they could adjust the intensity and demands of the curriculum to their child's energy level. Hence, if the child felt tired, he or she could do less work, and make up the work when he or she had more energy. A member check, with another group of parents and children, both with CIs and siblings, indicated they supported the themes from our study. The member check and use of a verification coder are techniques that strengthen the validity of study findings (Creswell 1998; Nabors and Liddle 2017). The use of art allowed the children to create as they completed their interviews and served as a prompt to help them reflect on their experiences and was positively received by children and parents. Art is a positive way to work with children, orient them to questions and elicit conversation about hospital experiences (Nabors and Liddle 2017).

Parents, children with illnesses, and siblings reported positive experiences with completing schoolwork while being at the hospital or Ronald McDonald House. For those who were in community schools, parents served as tutors and asked questions of teachers primarily through texting and emails. Parents value education for their children and maintaining school progress while in the hospital (Delloso et al. 2021; Uhm and Choi 2020). However, parents appreciated that teachers sent less work, focusing on the essential schoolwork needed to remain on par with classmates. Other research has indicated that children with CIs face exhaustion and may benefit from a reduced workload (Harden et al. 2020). Less information was available about sibling workload, and if a reduced workload was beneficial for them, and this remains an area for future research. Overall, results were positive, indicating that parents and children with CIs and siblings were keeping up with work and did not necessarily need hospital schools, which can add expenses for hospitals.

According to parents and children, teachers were sensitive to the needs of children with CIs. For instance, parents felt confident that they could ask for help from a teacher. Most reported the teacher was sensitive to the need to complete the ‘important work’ without extra work that was not needed for children to move ahead academically. This helped reduce parent and child stress, because they both believed the child could complete the work he or she was assigned. Interestingly, most children with illnesses and siblings felt that they could complete the work independently, asking their parents for help if they needed it. Being able to do schoolwork is part of the normal routine for children, and doing assignments while in the hospital with parents as tutors may help with fostering a sense of competency, accomplishment and normalcy, improving the child's feelings of wellbeing (Bravo et al. 2020; Ciucci et al. 2024; Hu et al. 2022). Most children with illnesses who attended community schools felt that they could easily return to the classroom routine when they went home.

There was a small subset of children with CIs and parents who did worry about reintegration into school. Siblings did not express these concerns, however. Typically, concerns focused on the school staff being able to manage the child's medical condition, fueled by worries from parents. Other research has noted this as well (Baker and Claridge 2022; Morales Ruíz et al. 2025). Children with illnesses and parents who are concerned would benefit from reintegration programming and connecting the medical team to school nurses and teachers to help with planning for education that incorporates knowledge about the child's medical condition (Carlton et al. 2021; Leite et al. 2023). Some parents shared that high levels of absenteeism can also be a source of stress, as children miss critical instruction and often face difficulties in catching up on assignments, and this sentiment has been echoed in other research (Barnett et al. 2020; Hu et al. 2022; Wikel and Markelz 2023). It will be important for health professionals to ask parents and children about school and reintegration to determine needs for reintegration planning and for special education services to meet needs related to the child's medical condition.

Children with CIs and siblings who attended community schools missed the social aspects of school when they were at the hospital or staying at the RMH (Byhoff et al. 2022; Spencer et al. 2023). They missed their friends at school and involvement in ‘specials’ like physical education classes as well as extracurricular activities. Assisting children in meeting with friends through online meetings and through texting and telephone calls may have improved their sense of connection to friends at school (Baskaran et al. 2022). A few children who were homeschooled were missing social connections and ensuring that they participate in social activities with peers can play an important role in their social development. There was a small subset of children who attended community schools who missed interacting with their teachers. Brief online meetings or telephone calls with teachers may enhance feelings of connection with teachers. Children who were homeschooled did not mention missing teachers, and they liked being able to study what they were interested in and liked being able to do homework at different times during the day—and not having to go to school for a traditional school day.

Most of the parents who homeschooled their children were positive about it. Several parents highlighted the benefits of homeschooling, noting the flexibility because they could tailor lessons to their child's interests and abilities. They often reported that they elected to homeschool so that they could care for their child's illness, feeling that teachers were unequipped to cope with the medical regimen and be attuned to their child's physical needs (e.g., fatigue, difficulties with concentration). Other researchers have indicated that parents assume a nursing or medical role with their children (Nygård and Clancy 2018). Homeschooling allowed some parents to ensure that they could work school into their family schedule when the child with an illness had many medical appointments. As mentioned, being absent from school has been a common barrier to school performance and participation for children with CIs (Barnett et al. 2020; Hu et al. 2022; Wikel and Markelz 2023). Parents were positive about curricula, which were not expensive, and for those who purchased curricula, they could find support if they needed help with teaching the materials. Others mentioned that they could find materials they wanted to teach and then find classes and experiences to supplement their teaching when they needed extra support. However, more information is needed about the curriculum available to parents and how they implement the curriculum. It is noteworthy that the high rate of parents homeschooling (71%) in our sample may reflect sampling bias due to the setting (e.g., RMH parents with access to time and resources). Further research with a larger and more diverse sample is needed to explore the benefits and drawbacks of homeschooling for children with CIs and siblings.

While homeschooling offers certain advantages, such as the flexibility to adapt lessons, it also presents challenges. One significant downside noted by parents is the loss of access to specialized support services typically available in traditional school settings, such as literacy interventions, speech and language therapy, and physical and occupational therapy. Furthermore, parents noted that homeschooling can limit opportunities for peer interaction and participation in extracurricular activities, such as sports and special classes. This may hinder a child's ability to form meaningful social connections and engage in the broader school community. However, most parents felt that they were able to find extracurricular activities to involve their children with peers. Two parents and children with CIs reported that the child experienced bullying related to his or her illness, and this resulted in the parents deciding to homeschool their child. Helping children who experience bullying through counselling the child and educating peers is important in these cases. Healthcare professionals should inquire about homeschooling and have referrals for therapies available, including referrals for counselling if the child experienced bullying. Further, healthcare professionals should inquire about child social functioning and be ready to discuss options like scouts, involvement in recreation centres, sports and community classes (art, dance, music) to improve social connections for children.

Several limitations may have influenced study findings and the generalizability of results for this study. The primary issues include significant selection bias from recruiting exclusively at one RMH, which captured only higher‐functioning families who could navigate enrolment in the RMH and continue with the child's schooling, which limits generalizability. The sample size was small, especially for the sibling group, and with a larger sample, more information might be discovered. Ensuring representation from children and families of colour in the sample would provide information about their educational needs. The single‐point‐in‐time data collection fails to capture the dynamic nature of educational needs throughout illness trajectories. Additionally, the absence of teacher perspectives represents a critical gap given their central role in educational continuity. Interviews investigated school functioning, which is important; however, it may have been beneficial to ask more questions about child emotional functioning. Learning more about school reintegration programming and support from school nurses could have provided more information about adjustment after hospitalization. Most parents were mothers (about 71%), and learning more about fathers' perspectives will provide a more nuanced view of how hospitalization impacts the nuclear family. Children with a variety of illnesses participated, which is common in research (e.g., Nabors and Liddle 2017). Our noncategorical (Stein and Jessop 1982) approach yielded important information about school functioning; however, in the future, it might be advantageous to understand school issues for children with different illnesses or who are facing significant medical procedures that may keep them out of school for long periods of time. The high homogeneity of the sample (94% White parents, 71% mothers) and the striking finding that 71% of families homeschool deserves deeper exploration. Additionally, the member check sample was a different group, which can be a methodological limitation. Hence, while the study addresses an important topic and provides valuable insights about successful educational continuity with teacher support, these methodological limitations and presentation issues need to be addressed to strengthen the manuscript's contribution to the field.

Results suggest ideas for policy and educational practice. First, in terms of policy, it may be advisable to consider shorter periods of time at school in combination with some time spent in homeschooling so that children with CIs who need this type of accommodation have opportunities to attend school. This may provide opportunities to capitalize on social interactions with peers in the school setting. If schooling occurs at home or in the hospital, teacher contact with parents and providing parents with educational materials is a resource for the family. Educational plans should address academic continuity, providing a plan for teacher involvement when children with CIs (and their siblings) are absent, providing teachers with a blueprint for maintaining contact and adapting assignments to facilitate school involvement during hospitalization or absences due to medical visits as well as develop plans for school re‐entry that consider child fatigue and recovery after hospitalization (Thompson et al. 2015; Wilkie 2012). Educational plans may be optimized using an approach that considers family stresses and resilience. When developing plans for the child with CIs or a sibling, family functioning should be considered. Patterson's (2002) Family Adjustment and Adaptation Response Model can guide planning as this model encourages considering family stresses, daily hassles and family members' strengths when coping with the stress when developing interventions.

Encouraging school nurses and teachers to learn about how to take care of the child with CIs from healthcare providers and parents may improve integration of children with CIs in the school setting (Carlton et al. 2021; Leite et al. 2023). Healthcare providers need to collaborate with school nurses to teach them to meet the needs of children with CIs so that they can return to community schools and have complex medical needs accommodated. Using technology through online meetings is one way to connect with nurses and teachers for this educational process. School nurses and teachers also may need to schedule time with parents in the classroom and meetings in the school clinic so that they can learn how parents meet the medical needs of children with CIs. In addition, healthcare providers, as well as teachers and parents, need to screen children with CIs to see if they have experienced negative interactions related to having a CI or being physically different. If bullying has occurred, it needs to be addressed in terms of helping the child who was bullied by ensuring safety and providing support for positive experiences and perhaps also by educating children at the school about a child's condition in terms of strengths and limitations and providing ideas for interacting positively with the child to support them at school.

Findings suggested that children with CIs and siblings were successful in completing work during hospitalization. Parents benefit from close collaboration with teachers, on an as‐needed basis, and a reduced workload focusing on key subjects, to ensure that their child is keeping up with schoolwork. Children may miss their friends, and finding ways to foster connections with friends and classmates during hospitalization may foster a sense of connection to school and improve emotional functioning (Miao et al. 2024; Preyde et al. 2017). Data for this study were collected over a short period of time, with a relatively small group of participants. Further research may provide different results and reveal more about stress related to hospitalization, yielding more data about positive and negative implications of homeschooling and success in completing schoolwork during hospital stays. Hospitalization may be both a source of stress and resilience, and the long‐term implications of repeated and extended hospitalizations on stress and resilience for children and parents should be explored. Areas for research may include the long‐term impact of hospitalizations on academic outcomes, peer engagement and emotional strain for children, parents and teachers. Homeschooling was an alternative for some families, and understanding the quality of this schooling and the long‐term results of homeschooling on child academic and social development will determine the long‐term effects of this educational experience on the academic success and social development of children with CIs and their siblings. In the future, it will be important to examine the academic and social needs of children with cognitive effects related to their medical conditions and children with CIs who have experienced difficulties, such as bullying in the school setting, to develop optimal educational programming and experiences for these children. Future research triangulating teachers, healthcare providers and family experiences will advance knowledge about their ideas for improving education during hospitalization and inclusion in the classroom.

## Author Contributions

L. N. and M. W. conceptualization. L. N., R. J., N. B., J. L. and M. W. data curation and project administration. R. J. and L. N. data curation and analysis. L. N., R. J., N. B., J. L. and M. W. writing. M. W., R. J. and L. N. review and editing.

## Ethical Statement

This research was approved by the institutional review board at the University of Cincinnati as nonhuman subjects' research.

## Conflicts of Interest

The authors declare no conflicts of interest.

## Data Availability

The data that support the findings of this study are available from the corresponding author upon reasonable request.
